# Necrotizing Autoimmune Myopathy: A Rare Variant of Idiopathic Inflammatory Myopathies

**DOI:** 10.1177/2324709617709031

**Published:** 2017-06-14

**Authors:** Noman Ahmed Jang Khan, Shaza Khalid, Saad Ullah, Muhammad Umair Malik, Samer Makhoul

**Affiliations:** 1 Temple University, Philadelphia, PA, USA; 2 Conemaugh Memorial Hospital, Johnstown, PA, USA

**Keywords:** muscle necrosis, regenerating muscle fibers, myopathy

## Abstract

Idiopathic inflammatory myopathies are an unusual group of myopathies with annual incidence of 1 in 100 000 people in the United States. Necrotizing autoimmune myopathy comprises only 16% of this group. It usually presents with severe proximal weakness, lower extremity weakness, and severe fatigue while very rarely does it present with dysphagia and respiratory muscle weakness. Statin use, cancer, and connective tissue disorder are the usual associated risk factors. Anti-signal recognition particle and 3-hydroxy-3-methylglutaryl-coenzyme A reductase are the 2 most common autoantibodies associated with necrotizing autoimmune myopathy. In this article, we present a very rare case of a 66-year-old male who presented with shortness of breath and dysphagia requiring intubation and ventilator support. Creatine kinase was 23 000, myoglobin was 7000, and ANA was positive. All other autoimmune and infectious workup including Lyme disease was unremarkable. Muscle biopsy turned out remarkable for necrotizing myopathy. No evidence of statin use, active malignancy, or connective tissue disease was found. He was treated with high-dose corticosteroids and a short course of intravenous immunoglobulin with very mild improvement in symptoms. Anti-signal recognition particle and 3-hydroxy-3-methylglutaryl-coenzyme A reductase could not be performed as the patient refused to pursue further medical testing. This is a very rare case of idiopathic inflammatory myopathy presenting with bulbar and respiratory muscle weakness requiring ventilator support.

## Introduction

Necrotizing autoimmune myopathy (NAM) is an unusual and rare subgroup of inflammatory myopathies. To date very few cases of NAM have been reported. Several risk factors including statin use (34%), malignancy (9.5%), and connective tissue diseases (4.2%) have been identified, whereas more than 50% were idiopathic in nature. Biopsy is required for diagnosis and shows pathognomic features of muscle necrosis and regeneration without any signs of inflammation. Treatment includes high-dose corticosteroids, early administration of intravenous immunoglobulin (IVIG), plasmapheresis, and immunotherapy with methotrexate, mzathioprine, rituximab, cyclophosphamide, and mycophenolate mofetil, addressing the underlying cause if any.

## Case

### History

A 66-year-old Caucasian male with no known past medical history presented with bilateral lower extremity weakness for 4 weeks. He initially went to a chiropractor and on no improvement sought medical attention. He denied any recent travels, camping in woods, sick contacts, recent flu-like illness. or diarrhea, rash, statin use, and tick bites. He also denied any vision loss, diplopia, active malignancy, or family history of any neurological disorder. The patient showed some improvement in muscle strength with corticosteroid therapy, refused muscle biopsy, and was discharged home on tapering dose of steroids. One week later he again presented to the hospital with dysphagia and shortness of breath. He was subsequently intubated and put on mechanical ventilator as he could not protect his airway and was started on high-dose corticosteroid therapy with IVIG.

### Physical Examination

The patient was alert, awake, and oriented to time, place, and person. Complete neurological examination was performed, which showed 1/5 power in both lower extremities, intact sensations, and absent ankle and knee reflex bilaterally. There was no rash, neck stiffness was not noticed, and there was negative Brudzinski’s sign.

### Differentials

1. Myositis
2. Dermatomyositis
3. Polymyositis
4. Necrotizing myopathy
5. Guillain-Barre syndrome
6. Lyme disease
7. Tick paralysis

### Labs

**Figure 1.**
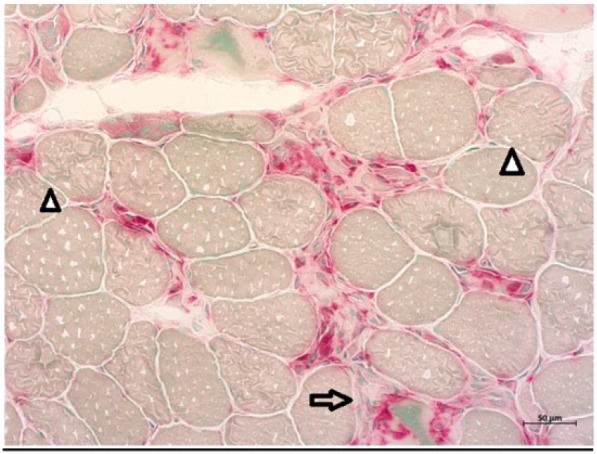
Acid phosphatase–stained sections of quadriceps femoris muscle showing necrotic muscle fibers invaded with macrophages (arrow) and regenerating muscle fibers (arrowheads).

**Figure 2.**
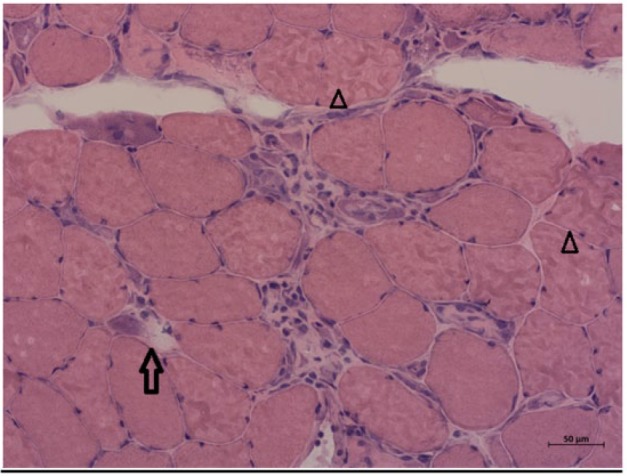
Hematoxylin-eosin–stained sections of quadriceps femoris showing various necrotic (arrow) and regenerating muscle fibers (arrowheads). Significant invasion of macrophages is also seen.

**Figure 3.**
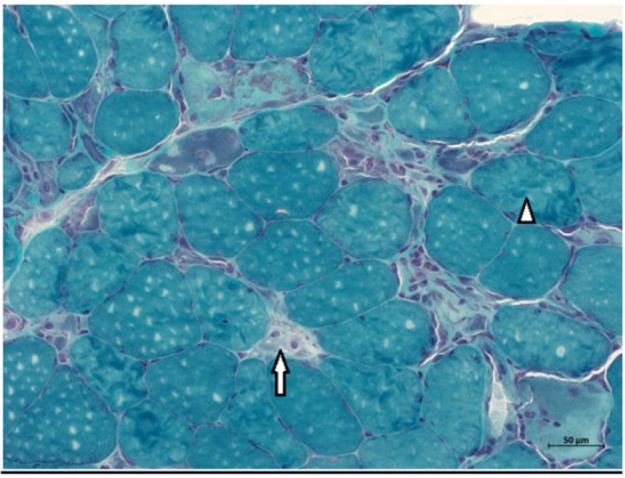
Trichrome-stained sections of quadriceps muscle demonstrate increased variability of muscle fiber size. Necrotic (arrow) and regenerating fibers (arrowheads) are present.

Initial laboratory workup revealed creatine kinase (CK) of 23 000, myoglobin of 7000, positive ANA, and negative testing for Lyme. All other relevant autoimmune workup was unremarkable (Table 1), and the patient was started on high-dose corticosteroid therapy. Electromyography (EMG) showed moderate peripheral polyneuropathy in the lower extremities. Magnetic resonance imaging of cervical, thoracic, and lumbosacral spine did not show any evidence of cord compression or radiculopathy, but diffuse myositis was noticed in all paraspinal muscles. This time he agreed to both lumbar puncture and muscle biopsy. Lumbar puncture was unremarkable, but muscle biopsy revealed very frequent necrotic and regenerative muscle fibers without any inflammatory process (Figures 1-3), findings classically consistent with necrotizing myopathy. Anti-signal recognition particle (anti-SRP) and 3-hydroxy-3-methylglutaryl-coenzyme A reductase (HMGCR) could not be performed as he refused to pursue further medical testing.

**Table 1.** Autoimmune Workup.

Antibody	Results
Anti-scleroderma	0
Anti-centromere	Negative
Cryoglobulin	None detected
ANA	1:640
Anti-Hu	None detected
SSB (LA) and SSB (RO)	None detected
Anti-Jo 1	1
Aldolase	>50
MuSK Ab	0.00

### Follow-up

The patient did not show much improvement in his respiratory status with corticosteroids and IVIG therapy. Eventually he got tracheostomy and a PEG tube and was transferred to a long-term rehabilitation facility on a short course of corticosteroids and IVIG. This is a very rare entity of myopathy that sometimes present with respiratory and bulbar muscle weakness requiring ventilator support and can prove fatal.

## Discussion

Idiopathic inflammatory myopathies (IIM) are a rare group of sporadic myopathies with annual incidence of approximately 1 in 100 000. NAM is a subgroup of inflammatory myopathies characterized pathologically by necrotic muscle fibers with absent or minimal inflammation. It comprises 19% of IIM, while dermatomyositis and nonspecific myositis makes 36% and 39% of the remainder group, respectively.^1^ The mean age of onset is 62 years with slight female predominance. NAM is idiopathic in almost half of the cases whereas other half is usually associated with statin use, malignancy, human immunodeficiency virus (HIV) infection, or connective tissue disease.^2^

Several risk factors have been identified. Statin use has so far been the largest identifiable risk factor for NAM, with atorvastatin and simvastatin being the most common ones.^3^ According to a recent study done at Mayo Clinic^4^ on 63 patients diagnosed with NAM on muscle biopsy, one third had documented evidence of statin use. Connective tissue disease, most commonly systemic lupus erythematous, have been found in some case studies. Malignancy was also found to be associated in almost 10% of cases of NAM with gastrointestinal malignancies being the most common.^5^

NAM generally presents with subacute, progressive proximal muscle weakness, lower extremity weakness, as well as weakness in other distal musculature, dysphagia, and dyspnea.^5^ Generalized weakness, myalgia, ambulatory difficulty, and decrease in appetite have also been present in some affected patients. Respiratory muscle weakness, as was seen in our case, is otherwise an uncommon presentation of this disease.^6^ On physical examination, higher mental and cranial nerve functions usually remain intact; however, muscle tenderness and reduced power have been found. No rashes have been described in patients with NAM.

Initial laboratory studies show grossly elevated CK. As compared to statin-associated myopathy where CK is in hundreds, CK in NAM is usually in thousands.^7^ EMG studies show abnormalities consistent with the irritable myopathic process. Muscle biopsy remains the gold standard for diagnosis, and histopathologic findings show presence of necrotic and regenerative myofibers. Oftentimes, there is an absence or minimal inflammatory infiltrate, although in the few cases when it has been present, there has been a predominance of macrophages.^8^ Of note, 2 markers, anti-SRP and anti-HMGCR Abs, have been described as having an association with NAM in about two thirds of cases. The pathogenicity between anti-HMGCR and NAM is uncertain, but patients with anti-HMGCR antibodies have usually a milder course of illness. On the other hand, seropositivity for anti-SRP did not predict any clinical severity, clinical course, or response to therapy.^9^ Our patient had CK levels of more than 20 000, positive ANA and aldolase, EMG consistent with irritable myopathy, and muscle biopsy significant for many scattered necrotic and regenerating fibers with absent inflammatory changes.

High-dose corticosteroids have generally been regarded as first-line treatment but most cases are refractory to conventional steroid monotherapy. Immunosuppressive therapy with methotrexate, azathioprine, rituximab, cyclophosphamide, and mycophenolate mofetil also has a role in the treatment of NAM. In addition, the early use of IVIG or plasmapheresis have been found associated with strength improvement and favorable outcome.^10^ Risk of relapse has been been found to be high during medication dose reduction or total discontinuation.^3^ Affected patients have been advised against the use of statin in future life as continuation has been found to be associated with worse outcomes.^7^ Moreover no significant differences in disease severity, outcomes, and response to therapy have been found among the different etiologic groups. Overall, the mortality in patients with IIM remains 2- to 3-fold higher than the general population, with cardiac, lung complications, cancer, and lung infections being the most common causes of deaths.^11^

## Conclusion

Necrotizing myopathy is a rare disorder of muscles with no known etiology in more than 50% of cases. Recognition of risk factors, identification of associated autoantibodies including SRP and HMGCR, timely muscle biopsy, and early aggressive immunotherapy are associated with improved outcomes.
